# Constrained acetabular liners in the instability of hip arthroplasty: what is its current role in revision surgery?

**DOI:** 10.1007/s00402-025-06110-5

**Published:** 2025-12-15

**Authors:** Luca Andriollo, Fabio Nesta, Alessandro El Motassime, Loris Perticarini, Rudy Sangalett, Francesco Benazzo, Stefano Marco Paolo Rossi

**Affiliations:** 1 Robotic Unit, UOC Ortopedia e Traumatologia, Fondazione Poliambulanza Istituto Ospedaliero, Brescia, Italy; 2 Catholic University of the Sacred Heart, Rome, Italy; 3 Agostino Gemelli University Polyclinic, Rome, Italy; 4 Istituto Universitario di Studi Superiori di Pavia, Pavia, Italy; 5 Department of Life Science, Health, and Health Professions, Link Campus University, Rome, Italy; 6 UOC Ortopedia e Traumatologia, Fondazione Poliambulanza Istituto Ospedaliero, Brescia, Italy

**Keywords:** Hip arthroplasty, THA revision, THA dislocation, Constrained acetabular liner, Hip arthroplasty instability

## Abstract

**Background:**

Dislocation of a total hip arthroplasty (THA) is a highly disabling complication following the implantation of primary and revision hip arthroplasties, as well as a prevalent reason for subsequent revisions. This study is designed to evaluate the survival rate, functional outcomes, and the reasons for further revision due to implant failure of Constrained Acetabular Liners (CALs).

**Methods:**

56 patients underwent hip revision surgery using a Constrained Acetabular Liner between June 2018 and December 2022 were retrospectively evaluated. Inclusion criteria consisted of age > 18 years, follow-up of at least 12 months, prior total hip arthroplasty or bipolar hemiarthroplasty, a history of recurrent implant dislocation, or, alternatively, the presence of a high risk of implant instability in hip revisions due to mechanical conditions.

**Results:**

The average age at the time of surgery was 72.4 years (SD 12.4). 55.6% of implants were performed for recurrent dislocation of THA, 8.9% for recurrent dislocation of bipolar hemiarthroplasty, 13.3% for aseptic loosening revisions, 4.4% for adverse reactions to metal debris revision procedures, and 17.8% for two-stage revision for periprosthetic joint infection. The average follow-up at the final evaluation was 32 months (SD 12.3). The survivorship of the implant was 88.9% at final follow-up. At the final follow-up: average HHS 77.4 ± 13.2; average WOMAC 31.4 ± 13.4; average OHS 32.1 ± 6.9; and average FJS-12 69.5 ± 19.6., and 65% showed excellent or good outcomes (HHS > 80).

**Conclusions:**

The CALs assessed in this study have shown satisfactory functional outcomes, even when compared with other anti-dislocation systems available on the market. Both cemented and uncemented solutions have shown a good survival rate in the mid-term. However, their use should be reserved for selected cases.

## Introduction

Total hip arthroplasty (THA) stands out as one of the most successful surgical interventions over the past five decades, achieving global recognition for its excellent outcomes and even being dubbed “the operation of the century” [1]. Despite its overall success, the persistent issue of instability poses a challenging and costly hurdle, significantly impacting patients’ quality of life [2]. Dislocation of a total hip arthroplasty (THA) is a relatively common and very disabling complication after the implantation of primary and revision hip arthroplasty [3–5]. Post-THA, the dislocation rate ranges from 1% to 10% in primary cases and climbs to 25% in revision THA, making instability a prevalent reason for revision THA, accounting for 17–33% of all revisions [6–9].

The risk of dislocation is multifactorial. The position of the cup plays a pivotal role in the stability of the hip. Historically, correct positioning was linked to adherence to the safe zone described by Lewinnek et al., with a reduced rate of dislocations within an inclination of 40° ± 10° and 15° ± 10° of anteversion [10]. However, today the importance of combined anteversion between the cup and the femur is emerging, rather than just the position of the acetabular implant alone [11, 12].

Recent studies on the rate of dislocation related to surgical technique indicates that after THA, the posterolateral approach is associated with a slightly higher dislocation rate at 1.1% when compared with the direct anterior approach at 0.7% and the lateral approach at 0.5% [13]. However, with no differences in revision rates or timing, recent studies attribute a reduced impact to the surgical approach compared to older literature [13, 14]. The dislocation rates turn out to be 2.7–8.3% for THA on acute hip fractures, thus higher [15].

Another fundamental factor is the restoration of the hip’s center of rotation, with respect for offset and length, which can reduce the risk of dislocation [16, 17]. Moreover, it is fundamental to evaluate the hip-spine relationships [18].

The natural history of THA dislocation often leads to less than ideal results for patients, persistently shadowed by the chance of ongoing instability and the need for further surgical interventions [19]. Moreover, the instability that occurs following revision THA poses an even more significant challenge and frequently becomes the primary reason for undertaking additional revision surgeries [20].

Dislocation may be managed through closed reduction, surgical reduction, or by undergoing a revision THA. The goal of revision surgery due to instability is to address the root causes of the instability. This could involve the revision of the acetabular cup or femoral stem, increasing the femoral head size, using different polyethylene, reducing extraarticular impingement [20]. In scenarios with recurrent hip dislocation or revision of THA with insufficiency of the gluteus muscles, the use of tripolar acetabular liners offers an alternative solution to help prevent further dislocations. This can be achieved through the use of dual mobility liners (DM) or constrained acetabular liners (CAL) [21, 22]. The use of CAL should be reserved for selected patients, with recurrent instability after the failure of other joint stabilization procedures, even through the use of implants with lesser degrees of constraint [21].

The primary goal of this research is to assess the survival rate and functional outcomes of Zimmer Biomet CALs (Trilogy Longevity Constrained Liner or Trabecular Metal™ Acetabular Revision System Cemented Constrained Liner; Zimmer Biomet, Warsaw, IN, USA) when used in treating recurrent hip arthroplasty dislocations. The secondary aim is to investigate the reasons for further revision due to implant failure.

## Materials and methods

A retrospective evaluation was performed on patients who underwent hip revision surgery using the Trilogy Longevity Constrained Liner (Zimmer Biomet, Warsaw, IN, USA) or Trabecular Metal™ Acetabular Revision System Cemented Constrained Liner (Zimmer Biomet, Warsaw, IN, USA) between June 2018 and December 2022. All surgical procedures were carried out by three experienced hip revision surgeons at a high-volume institution.

The patients enrolled in this study have been treated with anti-dislocation implants, which is indicated for conditions associated with a high risk of implant instability, especially in revision procedures. This includes patients with a history of previous dislocations, cognitive decline, neurodegenerative pathologies, abductor muscle weakness, and low functional needs.

Inclusion criteria consisted of age > 18 years, follow-up of at least 12 months, prior total hip arthroplasty or bipolar hemiarthroplasty, a history of recurrent implant dislocation, or, alternatively, the presence of a high risk of implant instability in hip revisions due to mechanical conditions (e.g., difficulties in restoring offset, abductor muscle incompetence due to adverse reactions to metal debris (ARMD), or periprosthetic infection, or oncological replacement). Patients with underlying patient-related conditions such as cognitive decline or specific neurodegenerative conditions (e.g., axonal neuropathy, Parkinson’s disease) were also included.

Patients who had undergone the use of a constrained liner as an anti-dislocation measure during the primary hip arthroplasty procedure, in elective or fracture conditions, were excluded.

Patient demographics were collected, as well as the reason for revision. In patients treated for recurrent dislocation, information was also gathered on the number of dislocations prior to the revision surgery, as well as the time elapsed between the primary hip arthroplasty procedure and the first dislocation. In patients with recurrent dislocation, the devices implanted during the primary hip arthroplasty were evaluated, particularly regarding the size of the femoral head.

Before the surgical procedures, a comprehensive preoperative evaluation of the patients was conducted. In addition to the initial clinical assessment, all patients underwent preoperative radiographic evaluation, including pelvic, hip, and lumbosacral X-rays for the assessment of both joint condition and spinopelvic relationships. In cases of recurrent dislocations, hip CT scans were performed, and in the presence of bone loss, CT scans were also conducted. For specific cases, such as patients with adverse reactions to metal debris (ARMD) or a history of periprosthetic joint infections (PJI), magnetic resonance imaging (MRI) was employed to examine soft tissue, especially the abductor musculature. Additionally, in cases where peripheral deficits were assessed during the clinical examination, neurophysiological studies were conducted, including electromyography and nerve conduction studies.

In all patients, the Trilogy Longevity Constrained Liner (Zimmer Biomet, Warsaw, IN, USA) or Trabecular Metal™ Acetabular Revision System Cemented Constrained (Zimmer Biomet, Warsaw, IN, USA) was employed as anti-dislocation system.

In patients with acetabular cup stability and compatibility with the Trilogy Longevity Constrained Liner, the constrained liner was implanted onto an existing Trabecular Metal™ Modular Acetabular Systems Shell (Zimmer Biomet, Warsaw, IN, USA). In patients who underwent revision of the acetabulum using the Trabecular Metal™ Acetabular Revision System Shell (Zimmer Biomet, Warsaw, IN, USA), the Trilogy Longevity Constrained Liner (Zimmer Biomet, Warsaw, IN, USA) was still utilized. In cases where acetabular revision was required along with the concurrent use of cages for bone loss, such as the Burch-Schneider™ Reinforcement Cage (Zimmer Biomet, Warsaw, IN, USA) or the LimaCorporate Acetabular Cage (LimaCorporate, San Daniele del Friuli, UD, Italy), the Trabecular Metal™ Acetabular Revision System Cemented Constrained (Zimmer Biomet, Warsaw, IN, USA) was employed.

In cases of severe bone loss, when deemed necessary, Trabecular Metal™ Acetabular Augments (Zimmer Biomet, Warsaw, IN, USA) or impaction bone grafting using allograft bone from a tissue bank were employed.

In all procedures, as they were revision surgeries, periprosthetic infection was ruled out through preoperative and intraoperative investigations, following the ICM 2018 criteria [23].

Patients underwent routine venous thromboembolism (VTE) prophylaxis with low-molecular-weight heparin for 5 weeks after hip revision treatment, or alternatively chronic anticoagulant therapy, and routine perioperative prophylactic antibiotic therapy involving Teicoplanin 800 mg. This infusion was administered once intraoperative samples had been obtained to rule out an unrecognized periprosthetic joint infection as the cause for the revision. Antibiotic therapy was then resumed and continued for up to 48 h following the surgical procedure, with dosages divided into two daily administrations of 400 milligrams each.

The postoperative rehabilitation protocol was not standardized for all patients, with some cases where full weight-bearing was not permitted. All patients, whether on partial weight-bearing in the initial days or allowed full weight-bearing from the outset, were prescribed crutches for the first 30 days. The exercises aimed at immediate joint mobilization in all planes while avoiding dislocating positions.

At the final follow-up, all patients underwent a retrospective clinical evaluation included patient-reported outcome measures (PROMs) such as the Western Ontario and McMaster Universities Osteoarthritis Index (WOMAC), the Harris Hip Score (HHS), the Oxford Hip Score (OHS) and the Forgotten Joint Score (FJS-12). In cases where a revision procedure after implantation of a constrained acetabular liner was performed, all relevant information regarding the reasons for revision and the details of the revision procedures was collected.

Radiographic follow-up examinations were performed at 3, 6, 12, and 24 months after the procedure, and then every 2 years.

The study was performed in accordance with the ethical standards in the 1964 Declaration of Helsinki and with the HIPAA regulation. The Institutional Review Board (IRB) of the author’s institution defined this study as exempt from IRB approval (retrospective study on a well-established surgical procedure and commercialized implant).

### Surgical technique

The patient is positioned in the contralateral decubitus position relative to the hip undergoing revision, with a posterior support placed at the level of the sacrum and an anterior support at the level of the anterior superior iliac spine (ASIS). Skin disinfection is carried out using 2% chlorhexidine digluconate, and subsequently, the sterile field is established with disposable drapes. The surgical approach employed is the posterolateral hip approach, involving a longitudinal skin incision at the level of the posterior two-thirds of the trochanter, and exposure of the piriformis tendon, triceps coxae, while preserving the joint capsule. There is the possibility of extending the incision according to the Wagner approach if necessary for stem removal.

#### Trilogy longevity constrained liner

The Trilogy Longevity Constrained Liner (Zimmer Biomet, Warsaw, IN, USA) was inserted in patients with: an existing Trabecular Metal™ Modular Acetabular Systems Shell (Zimmer Biomet, Warsaw, IN, USA) from a previous procedure; during the revision of a bipolar hemiarthroplasty where the Trabecular Metal™ Modular Acetabular Systems Shell (Zimmer Biomet, Warsaw, IN, USA) was placed; or with the Trabecular Metal™ Acetabular Revision System Shell (Zimmer Biomet, Warsaw, IN, USA) placed during a revision surgery after the removal of the previously present device.

**Fig. 1:**
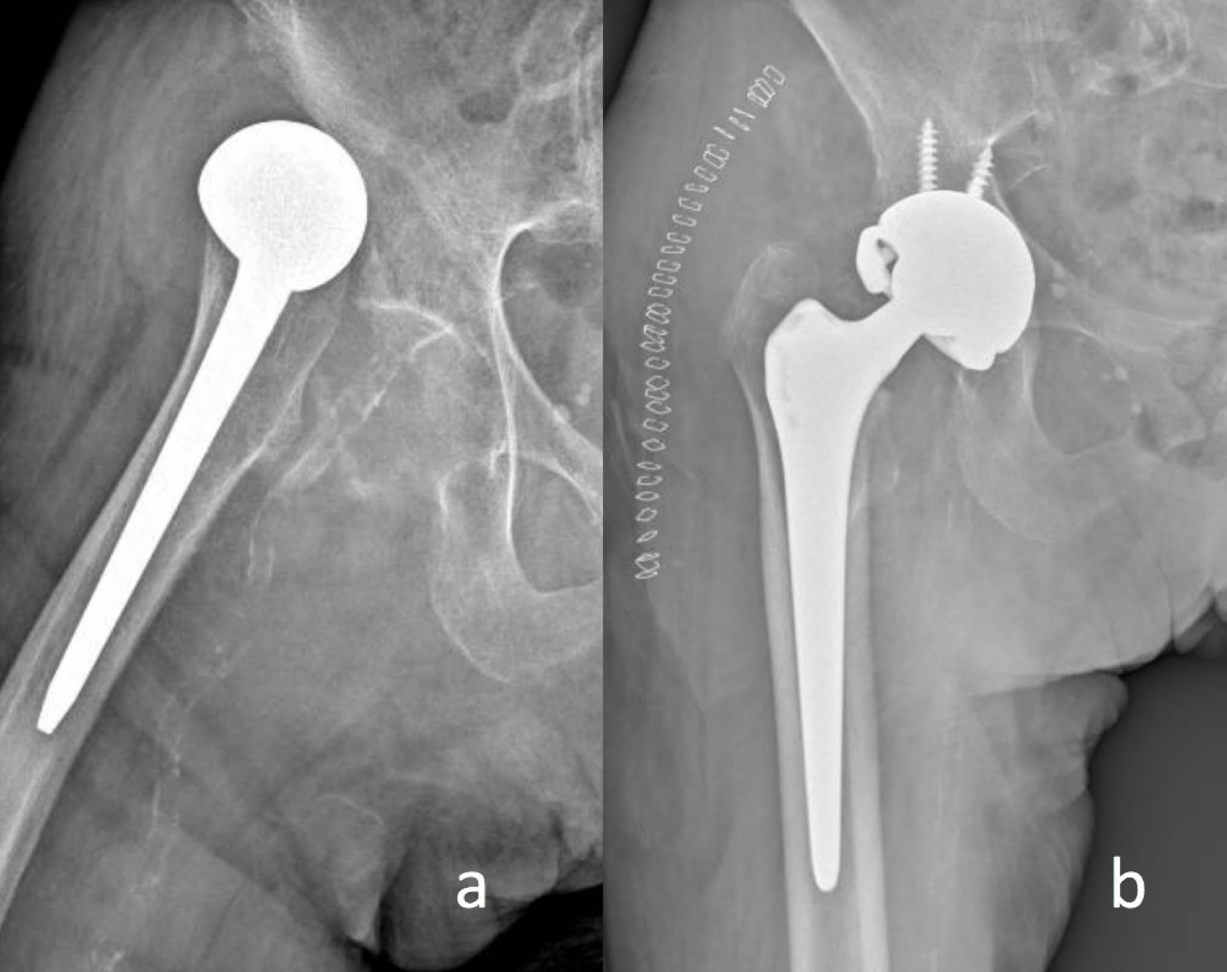
Recurrent dislocation of bipolar hemiarthroplasty (**a**), treated with revision surgery using a Trabecular Metal™ modular acetabular systems shell (Zimmer Biomet, Warsaw, IN, USA) fixed with screws and placement of a Trilogy Longevity Constrained Liner (Zimmer Biomet, Warsaw, IN, USA) (**b**).

With the shell positioned at the acetabular level, the procedure begins with the placement of the trial insert and verification of leg length and femoral offset. If no femoral revision is necessary, corrective measures are limited to replacing the femoral head. If a femoral stem revision is performed, this step should be carried out after the stem revision, before implanting the final components. At this point, a trial range of motion is also conducted. It is important to assess key ranges of motion, including: maximum flexion in neutral rotation; maximum internal rotation at 90° of flexion; full extension (but not hyperextension); and full external rotation in full extension. If premature impingement occurs, it is possible to modify the orientation of the constraining. Once the correct orientation is determined, it can then be replicated with the definitive liner. The next step is to place the Trilogy Longevity Constrained Liner, reduce the implant with final components, and lock it with the metal constraining ring, which is preliminarily placed on the femoral neck and subsequently fixed with a dedicated impactor. If possible, capsulorrhaphy and reinsertion of the external rotators are performed at the end of the procedure (Fig. 1).

#### Trabecular Metal™ acetabular revision system cemented constrained

After determining the necessary acetabular reconstruction implant, as previously explained, and conducting any required stem revision, the placement of the constrained implant is then performed.

The first step involves selecting a cemented constrained provisional liner size that matches the shell. Provisional liners are used to evaluate joint stability and the positioning of the face angle. The objective of the trial reduction is to identify the optimal rotational position for the constraining fingers to maximize the range of motion. This position can vary from patient to patient due to differences in anatomy and shell placement. Therefore, the liner’s position is adjusted to best meet the patient’s needs. Subsequently, a trial reduction is performed with the femoral stem and trial femoral head in place. After confirming leg length and femoral offset, a trial range of motion is conducted.

Once the ideal orientation of the trial insert is determined, the position of the center of the constraining fingers, indicated by an engraved line, is marked on the acetabular shell or cage. This mark serves as a reference point for replicating the placement of the constraining fingers during implantation.

Next, the COPAL G + V cement (Heraeus Medical GmbH, Wehrheim, Germany) is prepared. The size of the constrained liner varies based on the thickness of the cement used.

The liner is then positioned according to the plan, held in place until it sets, and excess cement is subsequently removed. Before reducing the femoral head into the liner, the constraining ring is placed at the level of the femoral neck. After reduction, the constraining ring is positioned correctly in engagement with the liner, and final implantation is executed using a dedicated impactor.

**Fig. 2:**
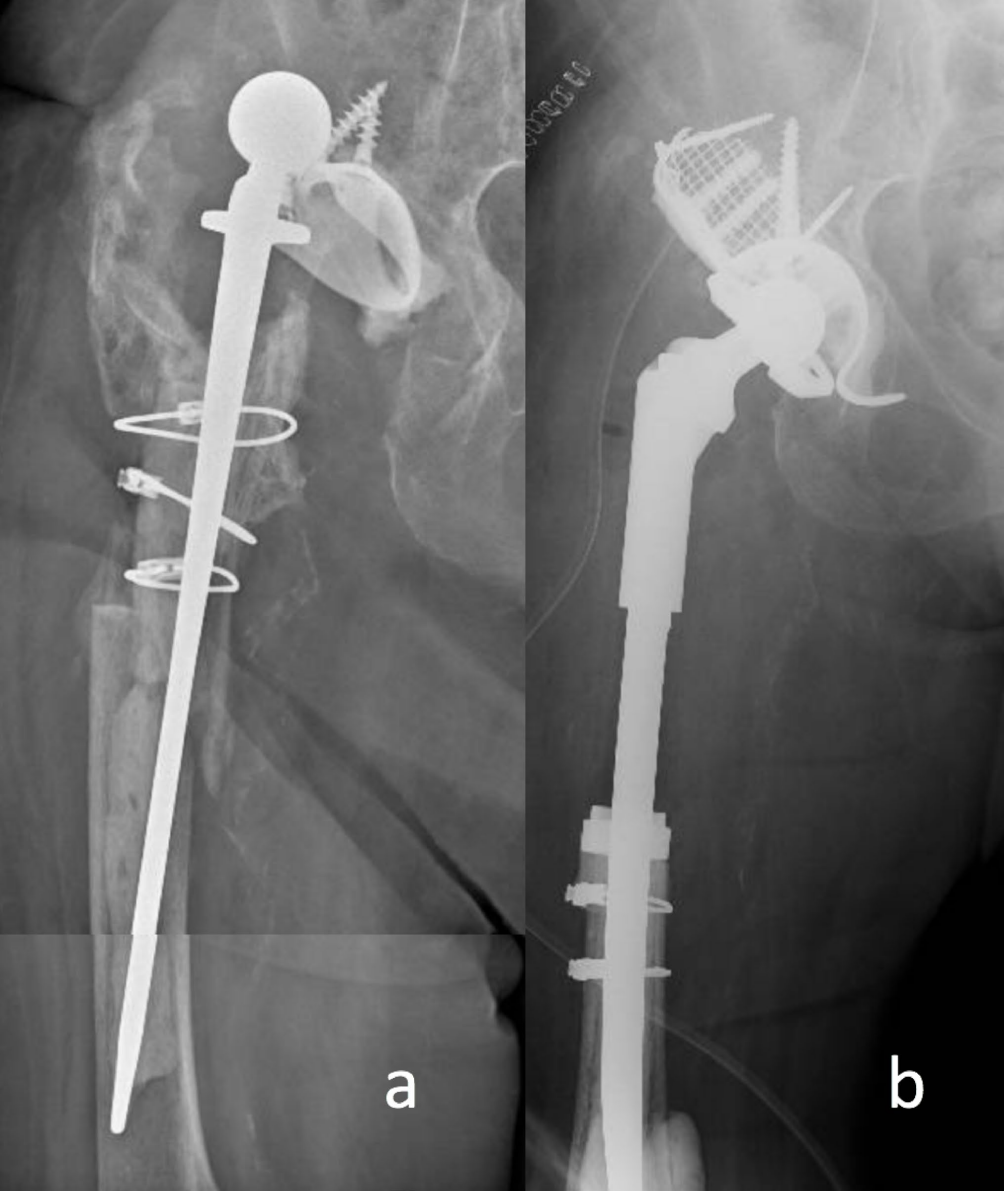
Periprosthetic fracture with severe femoral and acetabular bone loss (**a**), treated with a megaprosthesis stem and acetabular reconstruction using mesh, impaction bone grafting, LimaCorporate Metal™ Acetabular Revision System Cemented Constrained (Zimmer Biomet, Warsaw, IN, USA) (**b**).Acetabular cage (LimaCorporate, San Daniele del Friuli, UD, Italy), and cementation of a Trabecular

If possible, capsulorrhaphy and reinsertion of the external rotators are performed at the end of the procedure (Fig. 2).

## Statistical analysis

Statistical analysis was carried out with SPSS v18.0 (Chicago, IL, USA) by an independent statistician. Continuous variables were described using arithmetic average and SD (standard deviation). Categorical variables were described using frequency distributions and percentages. Survival analysis was conducted using the Kaplan-Meier methodology, with revision surgery as failure criteria. Confidence interval was set at 95%.

Level of Evidence III: retrospective cohort study.

## Results

Between June 2018 and December 2022, a total of 56 CALs were implanted in 56 patients. At the final follow-up, 7 patients were dead due to causes not related to the implant and 4 patients were excluded to lack of data. Consequently, the retrospective evaluation included a total of 45 patients, of which 14 were male (31.1%) and 31 female (68.9%).

All the implants were implanted on hip revision procedures, with 26 cases on the right side (57.8%) and 19 cases on the left side (42.2%).

Baseline demographics at the surgery time are displayed in Table 1. The average age at the time of surgery was 72.4 years (SD 12.4). 25 implants (55.6%) were performed for recurrent dislocation of THA, 4 (8.9%) for recurrent dislocation of bipolar hemiarthroplasty, 6 (13.3%) for aseptic loosening revisions, 2 (4.4%) for ARMD revision procedures, and 8 (17.8%) for two-stage revision for PJI.

**Table 1:** Demographics data.

**Patient population**	**Number**	**%**
Total no.	56	100
Died	7	12.5
Non traceable	4	7.1
Available	45	80.4
Indication	Number	%
Recurrent dislocation of total hip arthroplasty	25	55.6
Recurrent dislocation of bipolar hemiarthroplasty	4	8.9
Aseptic loosening	6	13.3
ARMD	2	4.4
PJI	8	17.8
Sex	Number	%
Male	14	31.1
Female	31	68.9
Age	Average (Y)	SD
	72.4	12.4
Side	Number	%
Left	19	42.2
Right	26	57.8
BMI	Kg/m^2^	SD
	23.7	47

In patients treated for recurrent dislocation, 21 (72.4%) experienced their first dislocation within 3 months following primary hip arthroplasty surgery. The average number of dislocations before undergoing revision surgery was 2.3 (SD 1.1). Among the 25 patients treated for recurrent dislocation of THA, 16 (64%) had a head size of 32 mm and 9 (36%) had a head size of 36 mm. In the 4 patients treated for recurrent dislocation of bipolar hemiarthroplasty, 2 (50%) had a head size of 22 mm and 2 (50%) had a head size of 28 mm.

All bipolar hemiarthroplasty surgeries were primarily indicated for proximal femoral fractures, while THA were performed in 32 cases (71.1%) for osteoarthritis and in 7 (15.6%) for fractures. In all revision scenarios without prior instability, the decision to use a constrained liner was due to the high risk of implant instability, whether caused by mechanical factors or neurodegenerative conditions.

In acetabular reconstruction, the Trabecular Metal™ Acetabular Revision System Shell (Zimmer Biomet, Warsaw, IN, USA) was used in 5 patients (11.1%), the Burch-Schneider™ Reinforcement Cage (Zimmer Biomet, Warsaw, IN, USA) in 2 (4.4%), and the LimaCorporate Acetabular Cage (LimaCorporate, San Daniele del Friuli, UD, Italy) in 4 (8.9%) cases. In patients undergoing acetabular revision with the Burch-Schneider™ Reinforcement Cage (Zimmer Biomet, Warsaw, IN, USA) and the LimaCorporate Acetabular Cage (LimaCorporate, San Daniele del Friuli, UD, Italy), a cemented constrained liner was implanted.

Consequently, out of a total of 45 patients evaluated, 39 (86.7%) were implanted with the Trilogy Longevity Constrained Liner (Zimmer Biomet, Warsaw, IN, USA) and 6 (13.3%) with the Trabecular Metal™ Acetabular Revision System Cemented Constrained (Zimmer Biomet, Warsaw, IN, USA).

In cases of severe bone loss, 5 patients (11.1%) required the use of impaction bone grafting with allograft bone, while in 2 patients (4.4%), Trabecular Metal™ Acetabular Augments (Zimmer Biomet, Warsaw, IN, USA) were employed. In 3 procedures (6.7%), the femoral component was also revised, involving the removal and replacement of the stem, using a monoblock stem in 2 cases and a modular stem in the remaining one.

**Fig. 3:**
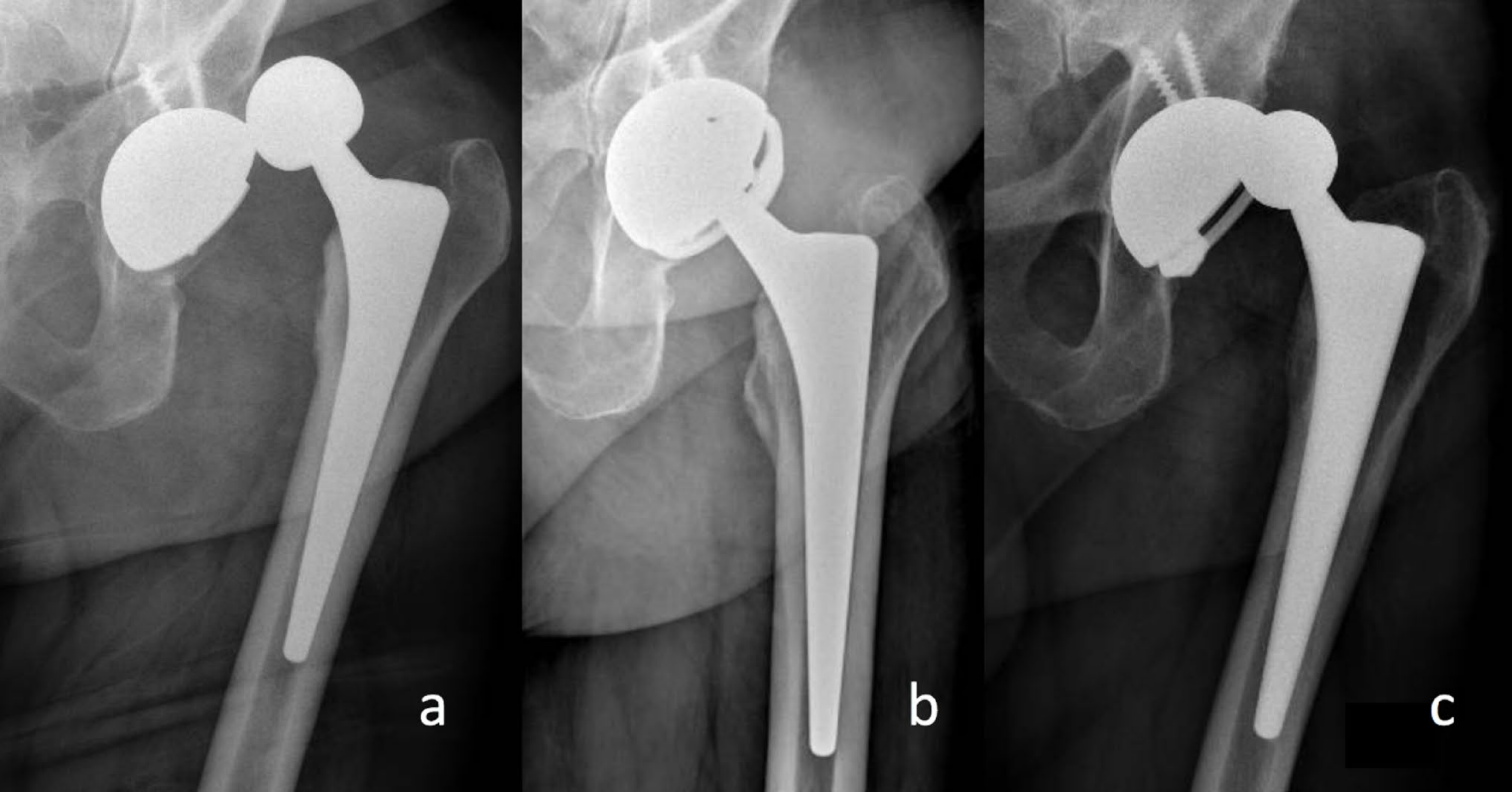
Recurrent dislocation of a primary total hip arthroplasty (**a**), treated with the placement of a Trilogy Longevity Constrained Liner (Zimmer Biomet, Warsaw, IN, USA) (**b**). Subsequent new dislocation, despite the constrained acetabular liner (**c**).

5 patients (11.1%) underwent revision surgery, and the average time to revision was 8.6 months (SD 7.8). 1 implant (2.2%) were revised due to periprosthetic joint infection, 2 (4.4%) due to constrained liner disassembly, 1 (2.2%) due to aseptic loosening and 1 (2.2%) due to periprosthetic fracture (Table 2) (Fig. 3).

**Table 2:** Revisions data.

**Revision**	**Cause of revision**	**Time to revision (mos)**
1	Infection	2
2	Constrained liner disassembly	3
3	Periprosthetic fracture	6
4	Constrained liner disassembly	11
5	Aseptic loosening	21

In 5 patients transfusion were needed in the post-op phase for an hemoglobin level below 8 g/dl.

**Fig. 4:**
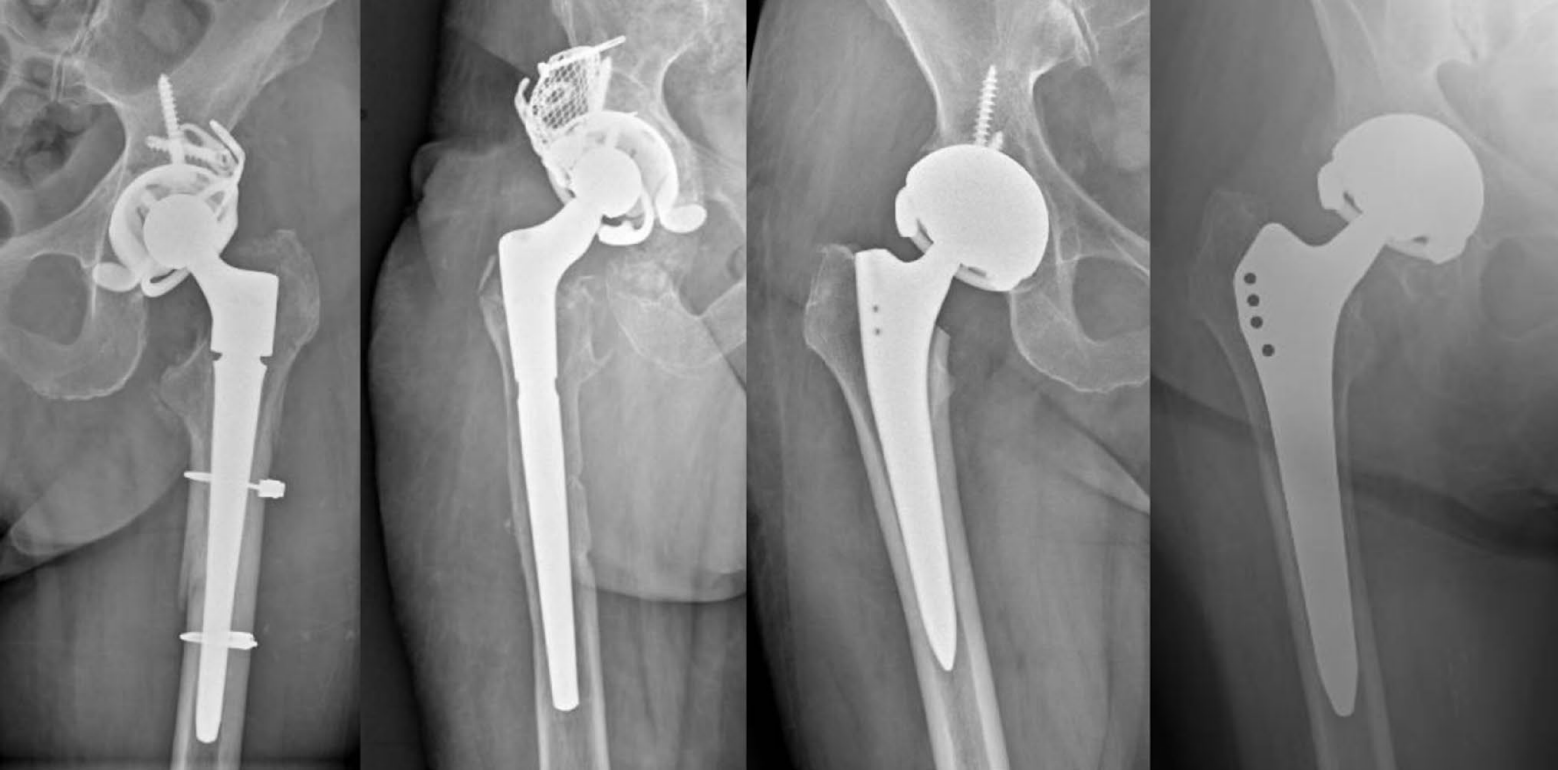
Series of X-rays at follow-up: some types of implants with constrained acetabular liners

Outcomes were assessed for 40 patients, after accounting for deaths, revisions and exclusion criteria. The average follow-up at the final evaluation was 32 months (SD 12.3). From the radiographic analysis, in patients with implant survival, no mobilizations or radiolucent lines were detected at the bone-implant interface (Fig. 4).

The survivorship of the implant was 88.9% at final follow-up.

The average HHS at final follow-up was 77.4 (SD 13.2). The average WOMAC score at final follow-up was 31.4 (SD 13.4). The average OHS was 32.1 (SD 6.9). The average FJS-12 at final follow-up was 69.5 (SD 19.6). At the final follow-up, 5 (12.5%) showed excellent outcomes (HHS > 90), 21 (52.5%) good outcomes (HHS: 80–89), 12 (30%) fair outcomes (HHS: 70–79), and 2 (5%) poor outcomes (HHS < 70) outcome. The data related to PROMs is reported in Table 3.

**Table 3:** Outcomes data.

**Available at the last FU**	**Number**	**%**
	40	88.9
Final FU	Average (mos)	SD
	32	12.3
Clinical outcome	Average (pts)	SD
HHS	77.4	13.2
OHS	32.1	6.9
WOMAC	31.4	13.4
FJS-12	69.5	19.6
Quality score HHS	No. of cases available	%
Excellent >90	5	12.5
Good 80 - 89	21	52.5
Fair 70 - 79	12	30
Poor < 70	2	5

## Discussion

The most important findings of this study are that the survival rate of Zimmer Biomet’s CALs at a mean follow-up of 32 months is 88.9%, which is higher than the failure rates reported in the literature. The primary indication for the use of CALs was recurrent dislocation, accounting for 68.9% of the cases. The study includes patients with CAL implants for both revision or reconstruction of the acetabulum and in a pre-existing shell. Furthermore, the PROMs assessed in terms of HHS, WOMAC, and FJS-12 are satisfactory, with 65% of patients reporting good to excellent outcomes. The results of this study are also consistent with the sample size of 56 patients, which is significant for a single-center study compared to other studies in the literature.

Despite the successes of hip prosthetics, instability remains a challenging problem that incurs high healthcare cost.

CALs operate by mechanically securing the femoral head, requiring greater force to dislodge it. The literature reveals a relatively high failure rate of these high-constraint systems with diminished functional outcomes [20, 24]. However, the constraint hip mechanism, while effective in femoral head capture, reduces the primary arc of motion in the hip. This capture is generally due to the liner’s circumferential elevation extending beyond the equator of the spherical head. The diminished arc of motion leads to earlier impingement. Furthermore, constrained liners can transmit elevated stresses across interfaces (liner cup, cup bone, liner cement, and cup cement), escalating the risk of accelerated polyethylene wear, liner disengagement, loss of fixation at implant-bone or implant-cement interfaces, potentially resulting in aseptic loosening and/or recurrent dislocation [20, 25–27].

Several studies are available that assess the implant survival and clinical outcomes of CALs in primary and revision hip arthroplasty, and in the revision of bipolar hemiarthroplasty with recurrent dislocations [28]. There is no consensus on the overall performance of these implants.

Therefore, the types of complications associated with this kind of implant, the cumulative incidence of hip dislocation after using CALs, and their clinical outcomes were assessed. It was also investigated whether the implant survival was in line with other alternative treatment methods such as DM and the use of large-diameter femoral heads.

The use of CALs has a relatively high complication rate compared to DM and the use of large-diameter femoral heads. It remains a rescue procedure for more complex patients affected by recurrent dislocations and instability of the implant. CALs should be used when the causes of instability have been correctly identified [21].

The primary indication reported in the literature for the use of CALs is their use in revision THA for the treatment of recurrent dislocation, followed by intraoperative instability in the context of revision, with percentages of 34.4% and 27% respectively according to a recent review published by Mancino et al. [21]. In particular, this systematic review reports an average rate of 4.9 prior dislocations, with the implantation of CALs in 62.1% into a retained well-fixed cup or in 37.9% following acetabular revision. The data from our study, however, report the implantation of CALs for recurrent dislocation in 68.9% of cases treated, with implantation after a dislocation rate of 2.3 (SD 1.1). In the patients enrolled in this study, the acetabular implant was retained in 73.2% of cases, excluding patients treated for the revision of bipolar hemiarthroplasty.

The complication rate reported in the systematic review published by Mancino et al. was 22.2%, with the most frequent complication being dislocation (9.4%), followed by aseptic loosening (5.2%) and infection (4.6%) [21]. Complications with a lower impact include periprosthetic fractures (3.4%), hematoma, seroma, and wound complications (0.5%), and nerve injuries (0.1%).

Our patients with complications reported major complications that required surgical revision in 11.1% of the cases at a mean follow-up of 2.7 years (SD 1.25). In comparison with the literature data, the complication rate was lower albeit with a shorter follow-up, as reported by Mancino et al. with 20.1% of complications requiring reoperation at a mean follow-up of 6.9 years [21]. Dislocation with disassembly of the constrained liner is the major cause in our data (4.4%) and in the literature (9.2%). In the literature, the all-cause reoperation-free survivorship after CAL implantation was 79.9%, with a more favorable rate reported in our data (88.9%).

However, there are conflicting data in the literature, with some studies reporting alarming complication rates. Specifically, numerous studies have reported overall complication rates higher than 30% at the final follow-up [29–33]. Some works reported rates even higher than 40% [34–36]. Only one study, published by Pattyn et al., reports complication rates in more than half of the treated patients, specifically 51.2% [37].

The overall complication rate, including revision surgery, was high, especially when compared to current coupling alternatives, such as DM cups and large-diameter femoral heads. Van Eecke et al. have documented an overall survivorship ranging from 88.9% to 100% for 3693 DM devices used in revision THA, with an average survivorship of 94.7% ± 3.5% across a follow-up period of 1.3–7.3 years [38]. They also noted a mean dislocation rate of 2.6% ± 2.5% and an acetabular loosening rate of 1.0% (ranging from 0 to 6.4%; SD, 1.5). DM cups exhibited dislocation rates from 0% to 1.4% following revision THA over short-to-midterm follow-ups, and contemporary DM designs have proven effective in reestablishing stability in cases of recurrent dislocation, with redislocation rates between 0% and 5.5% over short-to-midterm periods [25, 39, 40]. Large-diameter femoral heads have been shown to enhance the range of motion without impingement and to increase the jump distance [41, 42]. However, studies have yielded mixed results regarding the range of motion free from impingement as it relates to the diameter of the femoral head [43, 44]. Nonetheless, a notable decrease in dislocation rates post-revision THA has been observed with larger femoral heads (36–40 mm) in comparison to smaller ones (32 mm), at rates of 1.1% and 8.7% respectively [26]. Despite these benefits, large-diameter femoral heads have also been associated with several potential drawbacks, including an increase in volumetric wear, heightened stress on a thinner polyethylene liner and at the trunnion-head interface potentially leading to mechanical failure, local tissue reactions due to fretting corrosion when large metal heads are utilized, and possible impingement with the iliopsoas tendon [45–48].

CALs are utilized in multiple scenarios, predominantly for issues such as recurrent dislocation, insufficient soft tissue function with abductor mechanism deficiency, and neuromuscular disorders [49–51]. While these conditions are considered relative indications for the use of a CAL, the precise extent of soft tissue injury necessitating a constraining liner has not been explicitly determined. Moreover, the definition of “recurrent dislocation,” specifically the number of dislocations required before considering revision THA, remains not clearly defined. Nonetheless, CALs should generally be considered a last option, with an emphasis on first addressing any other potential causes of implant instability [20, 21, 38].

CALs are mainly reserved for salvage procedure due to multiple drawbacks. Fundamentally, CALs restrict the primary range of hip movement by capturing the femoral head, which can lead to impingement at the end of range of motion, potentially causing device failure [25]. This risk could shift the failure mode from dislocation to issues like loosening and wear [52].

The review by Mancino et al. highlights the design of a novel type of ACLS, the Trilogy Longevity Constrained Liner (Zimmer Biomet, Warsaw, IN, USA), which is examined in their study [21]. This CAL has been engineered to mitigate the issue of impingement through the inclusion of cutaway sections of the polyethylene, aiming to reduce impingement at common points of contact, such as anteriorly during flexion and internal rotation and posteriorly during extension and external rotation [34]. However, with this design, correct implant orientation becomes even more critical. A misaligned acetabular component, particularly if there is excessive abduction, retroversion, or anteversion, could exacerbate impingement and damage the liner. In studies that have evaluated this specific type of CALs, short-term outcomes have shown dislocation rates ranging from 3.7% to 12% [34, 53, 54].

The design of the cemented CALs evaluated in this study, the Trabecular Metal™ Acetabular Revision System Cemented Constrained Liner (Zimmer Biomet, Warsaw, IN, USA), follows the same principles.

Despite a relatively high complication rate associated with current treatment options, literature data indicate that patients treated with CALs have experienced considerable improvement in various functional scores [21]. Studies report an average HHS of 73.4 (range 59–85), which is consistent with the patients enrolled in our study who have an average HHS at final follow-up of 77.4 (SD 13.2) [30–32, 34, 35, 37, 53, 55–58]. In this study, the Oxford Hip Score (OHS) was 32.1 (SD 6.9), with literature data reporting an average of 36.9 (range 32–48.6.6) [37, 59, 60].

These findings imply that constrained liners can be an effective treatment option, enhancing patient function and outcomes, particularly for complex cases involving muscle weakness, neurological disorders, and repeated dislocations [21].

The limitations of this study include the relatively short follow-up, the heterogeneity of indications, and the retrospective design of this study as well as the absence of a control group. Nevertheless, this research, which reports satisfactory results both in terms of function and survival, highlights a high sample size for this type of CALs with an updated design, compared to other studies in the literature.

## Conclusions

The novel Zimmer Biomet Constrained Acetabular Liners assessed in this study have shown satisfactory outcomes, even when compared with other anti-dislocation systems available on the market. Both cemented and uncemented solutions have shown a good survival rate in the mid-term. It is crucial to remember that this type of solution should be considered in the presence of recurrent dislocations and instability of the implant, particularly due to deficits in the abductor musculature or neuromuscular disorders, as well as for patients with low functional demands. However, it should be utilized only after all other factors have been optimized, especially the orientation of the components, to minimize the risk of early mechanical failure and the necessity for revision surgery.

## Data Availability

Data are available in a separate data repository upon request.
